# The use of modified glove-like abdominal flap for reconstruction of contracture following burns of dorsal hand and fingers: A case report

**DOI:** 10.1016/j.ijscr.2022.106962

**Published:** 2022-03-29

**Authors:** Almahitta Cintami Putri, Puti Adla Runisa, Lisa Hasibuan, Ahmad Faried, Johanes Cornelius Mose

**Affiliations:** a Division of Plastic Reconstructive and Aesthetic Surgery, Department of Surgery, Faculty of Medicine, Universitas Padjajaran (FK UNPAD)-Dr. Hasan Sadikin Hospital, Bandung, Indonesia; b Graduate School of Biomedical Sciences, Doctoral Program, FK UNPAD, Bandung, Indonesia

**Keywords:** Hand contracture, Hand burn, Glove-like abdominal flap, Case report

## Abstract

**Introduction and importance:**

Burn contracture has been a challenge for its acquired functional disabilities and deformities. Surgical reconstruction poses a significant challenge for optimal aesthetic and functional improvement. Super thin abdominal skin pedicle flap can be used, but it has only one pedicle from one site of abdomen and needs tissue expander for a larger defect. The use of modified glove-like abdominal flap has been stated to be an option mainly for the use on acute hand burn. In this study, application of the modified glove-like abdominal flap was applied to contracture of dorsal hand and fingers.

**Case presentation:**

A 39-year-old male patient with severe contracted hand, eight-month post-burn injury presented at outpatient clinic Santosa Central Hospital in 2017. Multiple series of surgeries were performed on this patient, consisting of released contracture, defect closure using a modified “glove-like” thin abdominal flap, and flap separation.

**Clinical discussion:**

Abdominal flap has been the commonly used technique but has the disadvantage of being bulky. Glove-like abdominal flap, a subcutaneous layer plane flap, can be performed simply, safely, and briefly. It has been published mainly for reconstruction for acute burn hand injuries, not for burn hand contracture.

**Conclusion:**

The use of modified glove abdominal flap technique for reconstruction in hand burn contracture gives a satisfactory result in terms of functional and aesthetic outcome and can be an option in reconstruction in contracted dorsal hand and fingers.

## Highlights

• Abdominal flap has been used to be an option for hand burn reconstruction but has the disadvantage of being bulky.
• Glove-like abdominal flap has been published mainly for reconstruction for acute burn hand injuries, not burn hand contracture.
• Glove-like abdominal flap is an easy technique, safely, briefly, not bulky, and give better functional aesthetic results.
• Modified glove abdominal flap technique can be an option in reconstruction in contracted dorsal hand and fingers.

## 1 Introduction

Burn patient has a big portion of incidence and cause tremendous morbidities and mortalities [1]. The hands account for less than 5% of total body surface area, but despite the small percentage, burns affecting the area are considered severe injuries, often resulting in significant physical, and psychological requiring referral to a specialized burn center [2].

Regardless of improvement in the management of burn patients, late complications such as contracture will become a burden and impact the quality of life [3]. Reconstruction has always been a challenging aspect of burn surgery, sometimes requiring multiple staged procedures to achieve the acceptable functional and aesthetic results [4].

The principle in burn reconstructive surgery of contracted hand is divided into two basic steps; release the contracture and resurface the defect [5]. It is a challenge for plastic surgeons to cover the defect and concern to functional and aesthetic aspects [6]. The abdominal flap has been the commonly used technique but has the disadvantage of being bulky [7]. Super thin abdominal skin pedicle flap can be used, but it has only one pedicle from one site of the abdomen and needs a tissue expander for a larger defect [8]. The tissue expander can lead to complications such as failure, infection, and being exposed [9]. The technique using a thin glove-like abdominal flap, a subcutaneous layer plane flap, can be performed simply, safely, and briefly. It has been published mainly for reconstruction for acute hand burn injuries [10]. The novelty of this case report is the use of a glove-like abdominal flap on the reconstruction of burn contracted dorsal hand and fingers.

## 2 Materials and methods

This case report has been reported in line with the SCARE 2020 criteria [11] and has been approved by our ethics committee No:863/UN6.KEP/EC/2021. Written informed consent was obtained from the patient for publication of this case report and accompanying images. A copy of the written consent is available for review by the Editor-in-Chief of this journal on request.

## 3 Case presentation

A 39-year-old, male, Sundanese, coal mine worker and the right-handed patient presented at outpatient clinic Santosa Central Hospital Bandung Indonesia in 2017, for a complaint of hand stiffness due to blast injury scars of hot coal 8 months before hospital admission. Several debridement surgeries were performed for debridement of non-viable tissue, and the patient received wound treatment and went through medical rehabilitation in a rural hospital. The patient has no drug history, family history, and psychosocial history including smoking.

**Fig. 1:**
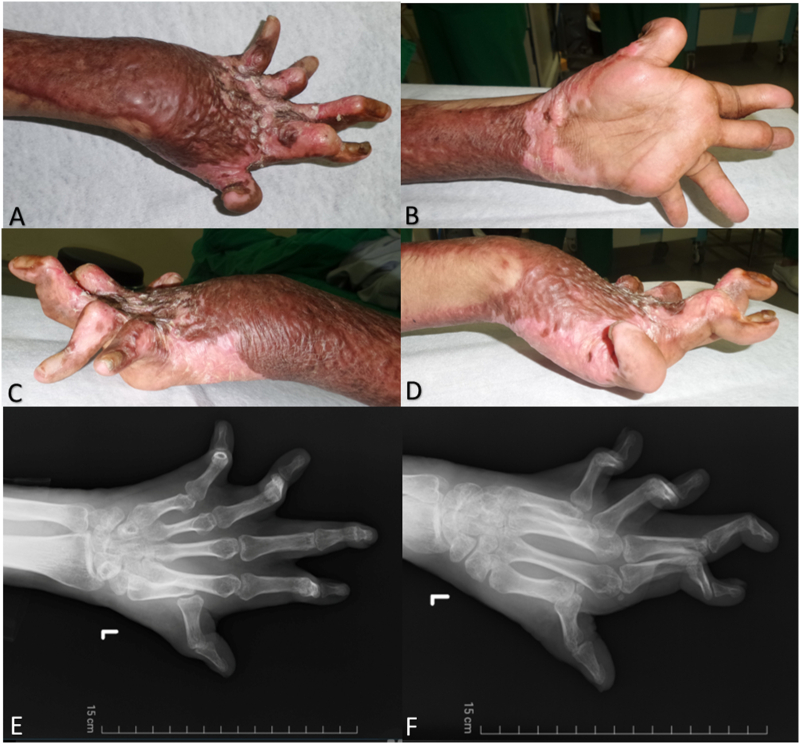
Clinical picture of the upper left extremity of the patient at first presentation. The left dorsal hand had extensive scars, forming swan neck deformity on the middle finger and Boutonnières deformities on the other fingers. All five digits were hyperextended at the MCP joint. The picture at the dorsal view (A), palmar view (B), lateral ulnar view (C), lateral radial view (D), and X-ray (E-F).

However, despite the tremendous time spent on extensive and time-consuming rehabilitation, the patient presented to the outpatient clinic with the presence of late complication of scars formation on his dorsal hand and fingers, causing severe contracture. The patient had burn scars on both upper extremities. The left dorsal hand had extensive scars, forming swan neck deformity on the middle finger and Boutonnières deformities on the other fingers (Fig. 1). All five digits were hyperextended at the MCP joint with type III Graham classification [12], demonstrating dorsal subluxation at the joints. Hand impairment was analyzed with all available data, including the range of motion, strength, and basic hand function (Table 1 and Table 2). The sensation of the hand was normal.

**Table 1:** Range of motion of the left hand at first presentation: joints of interphalangeal (IP), proximal interphalangeal (PIP), distal interphalangeal (DIP), metacarpophalangeal (MCP). * all firm end feel.

Joint	Flexion ROM	Extension ROM	Power
IP 1	−30° to −30°	30° to 30°	1
PIP/DIP 2	40° to 40°/−10° to 10°	−40° to −40°/10° to 10°	1
PIP/DIP 3	−10° to −10°/30° to 30°	10° to 10°/−30° to −30°	1
PIP/DIP 4	90° to 90°/−10° to −10°	−90° to −90°/10° to 10°	1
PIP/DIP 5	90° to 90°/−10° to −10°	−90° to −90°/10°-10°	1
MCP	−40° to −40°	40° to 40°	1
Wrist	10° to 10°	−10° to −10°	1

**Table 2:** Basic hand function at first presentation.

Basic hand function	Ability to perform
Pinching	No
Keying	No
Grasping	No
Power grip	No
Tripod	No

The patient was prepared for stage 1 surgical intervention. The supine position was performed and a tourniquet was used on the arm. The left hand was operated with an extensive excision of hypertrophic scars, exposing all deep structures of the dorsal surface of the hand. The extensor tendons of the digits and the wrist were shortened due to the contracture. Gradual stretching of the tendons was preferred overcutting and lengthening. The collateral ligaments were released and pericapsular dissections were required to obtain full release. In MCP joint extension contractures, capsulotomy was done, releasing the contracted collateral ligaments. Pockets fashioned on the volar side for the base of the proximal phalanges to glide down the head of the metacarpals [13]. All joints on all digits were repositioned to the closest possible anatomic position, maintained by an intramedullary 1.5 mm K-wire fixation for 3 weeks.

**Fig. 2:**
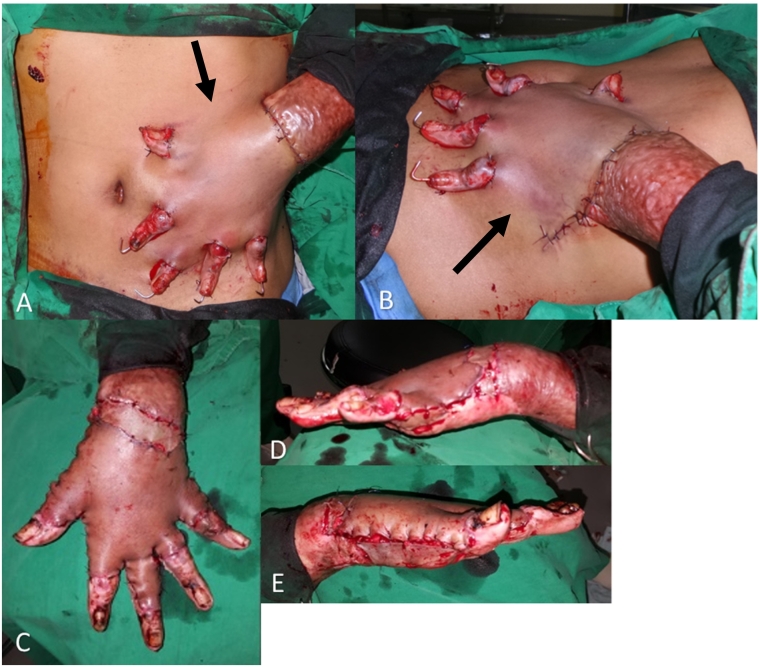
Clinical picture post hand inserting into the created pocket of the glove-like abdominal flap (arrowheads), a subcutaneous flap (A-B) and separated in three weeks later(C-E).

The contracture released hand was put in a comfortable and anatomically well positioned on the abdomen. A random pattern of a thin glove-like abdominal was designed and incised. A pocket was created by dissecting through a subcutaneous plane that preserves the subcutaneous vascular networks to make a thin flap. In the setting of the hand in the pre-prepared pocket accordingly (Fig. 2) and stitch the skin with a 4.0 non-absorbable nylon, (dermalon® Covidien US, Monofilament Nylon 4/0, Reverse Cutting 19 mm, 3/8 Circle). Post-operative instruction was the arm position being immobilized with splint and bandage. The patient could comply, and tolerate post-operative instruction.

Three weeks afterward, a stage 2 operation was performed. The hands were taken out with the preservation of the subcutaneous flap. The donor site and remaining raw surfaces were covered with split-thickness skin grafts. The palmar skin being in contact with the raw surface bed of the abdomen was macerated and healed by itself. This glove-like abdominal flap was the modified technique for the reconstruction of contracted dorsal hands and fingers. All the procedures were done by the first author, a plastic surgeon.

## 4 Results

**Fig. 3:**
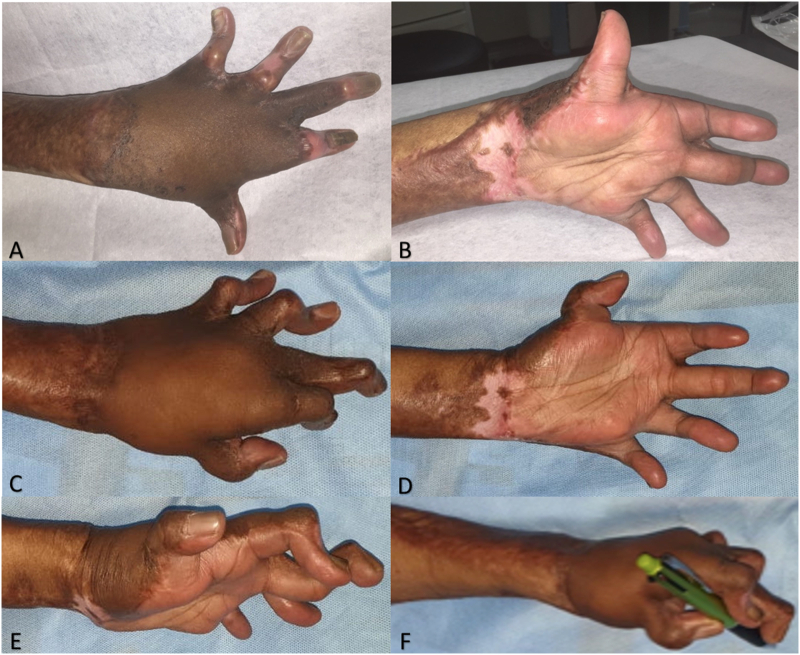
Clinical picture of the hand 3 months post-reconstruction: dorsal view (A) and palmar view (B). Clinical picture of the left hand, 2 years post reconstruction: dorsal view (C), palmar view (D), lateral view (E), and ability to pinch (F).

After follow-up at 3-month and 2-year post-surgery, with a significant improvement in the basic function of the hand (Fig. 3). After 2 years, even though the release of the contracture was not followed by the hand's anatomic structure before the incident, the patient was able to return to his daily activity and to work (Table 3, Table 4). No significant complication was faced after the procedure and the patient perspective was satisfied with the result.

**Table 3:** Range of motion of the left hand at 2-years post-surgery: joints of interphalangeal (IP), proximal interphalangeal (PIP), distal interphalangeal (DIP), metacarpophalangeal (MCP). * all firm end feel.

Joint	Flexion ROM	Extension ROM	Power
MCP-IP 1	0° to 20°/70° to 90°	0° to 10°/−70° to −90°	3
PIP/DIP 2	90° to 90°/−10° to −10°	−90° to −90°/10° to 10°	3
PIP/DIP 3	90° to 90°/−10° to −10°	−90° to −90°/10° to 10°	3
PIP/DIP 4	90° to 90°/−10° to −10°	−90° to −90°/10° to 10°	3
PIP/DIP 5	90° to 90°/−10° to −10°	−90° to −90°/10° to 10°	3
MCP	0° to 40°	0° to 0°	3
Wrist	0° to 20°	0° to 0°	3

**Table 4:** The basic function of the hand at 2-years post-surgery.

Basic hand function	Ability to perform
Pinching	Yes
Keying	Yes
Grasping	Yes
Power grip	No
Tripod	Yes

## 5 Discussion

Post-burn contracture has a significant impact on the quality of life in patients. It is not only on physical functioning but also complained of pain and vitality [3]. Reconstructive procedures can considerably improve the function of the hands. A suitable choice of procedures followed by controlled physiotherapy can be a help for a burns survivor [13]. Multiple surgical reconstructions are often necessary and pose a significant challenge for aesthetic and functional improvement [14].

A classification by Graham et al. [12] divided the metacarpophalangeal (MCP) joint extension contractures as type I: digits demonstrated greater than 30 degrees of MCP flexion with the wrist fully extended and scarring limited to the skin; type II: digits demonstrated less than 30 degrees of MCP flexion with the wrist maximally extended and involved skin to deeper structures such as MCP joint capsule; type III: digits were fixed in greater than 30 degrees of MCP hyperextension and has articular incongruity. This classification has prognostic utility and improvement seen in 95% of type I contractures, 73% of type II contractures, and only 47% of type III contractures. The presented patient, being classified as type III, was estimated to have a 47% chance of improvement [12]. Surgical steps in this situation are as follows: maximal release of the contracture; skin graft (split or full-thickness skin graft), choices of a wide range of flap; and post-operative physiotherapy, as well as serial splinting to obtain a further range of flexion motion. In addition, the use of K-wires is always recommended for 2 to 4 weeks post-operatively, as plaster alone is usually unable to maintain an adequate position [12]. Even though a second release might be needed, the technique is quite successful [14].

It is suggested that flap coverage was associated with a shorter time to healing, fewer procedures, and better functional outcomes than K-wire and grafting [15]. The abdominal flap has been the commonly used technique but it has the disadvantage of being bulky [7], [10]. A study by Woo and Seul reports coverage of corrected severe post-burn with various fasciocutaneous flaps, with a good flap survival and good functional gains in all but one hand [16]. Other types of flap such as regional island flaps [17], distant groin flap [18], and even free flaps [19] have been used for the reconstruction of hand contractures. All of the mention above are bulky flaps and takes a long time to perform.

A study performed by Gousheh in 2008 [8] presented a technique of super-thin skin abdominal flap for the treatment of dorsal hand hypertrophic burn contracture in 34 patients using the procedure. The overall functional and aesthetic results were evaluated as having good outcomes, without relapse of contracture, and the reconstructed skin was aesthetically similar to the surrounding skin in terms of laxity and color match, with a good scar. Super thin abdominal skin pedicle flap has only one pedicle from one site of the abdomen and needs a tissue expander for a larger defect [8]. The tissue expander adds cost and can lead to complications such as failure, infection, and being exposed [9]. Techniques using thin glove-like abdominal flap in subcutaneous layer plane have been published mainly for reconstruction for acute hand burn injuries. It can be performed simply, safely, and briefly [10]. This study serves as a benchmark for the application of the glove-like abdominal flap in our patient.

In 1965, an initial basic principle of the glove hand, differently named as “*crane flap*”, was described by Millard [20]. The concept consists of making a subcutaneous pocket in the patient's abdominal wall or groin region, cut like a glove where the patient's debrided hand is inserted. The hand will be taken out 2 to 3 weeks afterward with the preservation of the subcutaneous flap. Only a few reports appear after the appearance of this procedure in 1969, but the great potential is seen with the conjunction in the use with deep hand burns that exposed tendons and joints [20]. Most studies applied this method on early management in burn hand reconstruction, but very few in late contracture [10], [21].

Although our primary goal is to restore maximal function in the injured hand, we must also do everything we can to assist the patient in learning to live with the residual problems that we cannot make better. In our first experience in using the glove-like abdominal flap, we thought that the use of the technique was easy and reproducible, especially for non-experienced surgeons.

The author finds that the technique produces a thin flap with a high chance of flap survival because it has multiple skin and subcutaneous pedicles, therefore the blood supply is safe. For long observation follow-up, it has smaller risk of recurrence than using skin grafts. Even though, difficulty was found in the setting of the wired hands into the prepared pocket. The disadvantage of this technique was the 3 weeks required uncomfortable immobilization by the patient, which should be accompanied by a certain level of compliance. Also, the palmar skin being in contact with the raw surface bed of the abdomen was macerated. No significant complication was faced after the procedure and basic function of the hand.

The glove-like abdominal flap is an easy technique to be adapted, survive safely, performed briefly, and can be used as a reliable modality in the treatment of dorsal hand and finger burn contractures. The presented reconstructive technique favors all functional advantages of other flaps, being thick, preventing full range of motion, and lacking the drawbacks of skin grafts. Furthermore, from the aesthetic point of view, the reconstructed skin is not bulky and appears similar to the skin of the rest of the extremity, with a good color match, laxity, and suppleness.

## 6 Conclusion

The primary goal in hand reconstruction is the restoration of maximal function of the injured hand, even though the objective of obtaining a good aesthetic result should also be put into account [6], [13]. The use of modified glove abdominal flap technique for reconstruction in hand burn contracture gives a satisfactory result in terms of functional and aesthetic outcome and can be an option in reconstruction in contracted dorsal hand and fingers.

## Sources of funding

None.

## Ethical approval

Obtained.

## Consent

Written informed consent was obtained from the patient for publication of this case report and accompanying images. A copy of the written consent is available for review by the Editor-in-Chief of this journal on request.

## Author contribution

First author as study concept, design, data collector, operator, analysis, and editing; second author as study concept, data collector, analysis, and editing; other authors as study design, interpretation, analysis, and editing. The authors thank Istingadah Desiana, MD, a Physiatrist, for her contribution and support during this case report.

## Registration of research studies

Not applicable.

## Guarantor

Almahitta Cintami Putri, MD.

## Provenance and peer review

Not commissioned, externally peer-reviewed.

## Declaration of competing interest

The authors report no conflicts of interest. The authors alone are responsible for the content and writing of this article.
